# Direct Observation of Treatment Provided by a Family Member as Compared to Non-Family Member among Children with New Tuberculosis: A Pragmatic, Non-Inferiority, Cluster-Randomized Trial in Gujarat, India

**DOI:** 10.1371/journal.pone.0148488

**Published:** 2016-02-05

**Authors:** Paresh Vamanrao Dave, Amar Niranjan Shah, Pankaj B. Nimavat, Bhavesh B. Modi, Kirit R. Pujara, Pradip Patel, Keshabhai Mehariya, Kiran Vaman Rade, Soma Shekar, Kuldeep S. Sachdeva, John E. Oeltmann, Ajay M. V. Kumar

**Affiliations:** 1 Department of Health and Family Welfare, Government of Gujarat, Gandhinagar, Gujarat, India; 2 WHO country office for India, New Delhi, India; 3 National Tuberculosis Institute, Government of India, Bangalore, India; 4 Central TB Division, Ministry of Health and Family Welfare, Government of India, New Delhi, India; 5 U.S. Centers for Disease Control and Prevention, Atlanta, Georgia, United States of America; 6 International Union Against Tuberculosis and Lung Disease, South-East Asia Regional Office, New Delhi, India; The George Washington University School of Medicine and Health Sciences, UNITED STATES

## Abstract

**Background:**

The World Health Organization recommends direct observation of treatment (DOT) to support patients with tuberculosis (TB) and to ensure treatment completion. As per national programme guidelines in India, a DOT provider can be anyone who is acceptable and accessible to the patient and accountable to the health system, except a family member. This poses challenges among children with TB who may be more comfortable receiving medicines from their parents or family members than from unfamiliar DOT providers. We conducted a non-inferiority trial to assess the effect of family DOT on treatment success rates among children with newly diagnosed TB registered for treatment during June–September 2012.

**Methods:**

We randomly assigned all districts (n = 30) in Gujarat to the intervention (n = 15) or usual-practice group (n = 15). Adult family members in the intervention districts were given the choice to become their child’s DOT provider. DOT was provided by a non-family member in the usual-practice districts. Using routinely collected clinic-based TB treatment cards, we compared treatment success rates (cured and treatment completed) between the two groups and the non-inferiority limit was kept at 5%.

**Results:**

Of 624 children with newly diagnosed TB, 359 (58%) were from intervention districts and 265 (42%) were from usual-practice districts. The two groups were similar with respect to baseline characteristics including age, sex, type of TB, and initial body weight. The treatment success rates were 344 (95.8%) and 247 (93.2%) (p = 0.11) among the intervention and usual-practice groups respectively.

**Conclusion:**

DOT provided by a family member is not inferior to DOT provided by a non-family member among new TB cases in children and can attain international targets for treatment success.

**Trial Registration:**

Clinical Trials Registry–India, National Institute of Medical Statistics (Indian Council of Medical Research) CTRI/2015/09/006229

## Introduction

Directly observed treatment (DOT) is a supportive mechanism where a provider directly observes a patient consuming their medication to ensure treatment adherence and is a core component of the strategy recommended by the World Health Organization (WHO) to treat tuberculosis (TB). A classic study from Chennai, India showed that supervised domiciliary care was as effective as hospital-based care in achieving treatment success. Given the resource constraints and costs of hospitalizing TB patients and patient-inconvenience associated with prolonged hospitalization, supervised, ambulatory care (also referred to has DOT) became the standard of care [1, 2]. Researchers conducted randomized trials during the late 1950s and demonstrated similar patient outcomes for sanatoria-based and domiciliary based treatment, thus paving the way for domiciliary treatment and DOT [2, 3]. Although widely accepted, DOT has been the subject of much debate [4–9]. Concerns revolve around cost effectiveness of the intervention, patient convenience, patient privacy, and the effect on treatment outcomes [10–13]. Numerous studies have found no difference in treatment outcomes achieved under self-administered treatment versus DOT [8, 14]. However, the validity of these studies has been debated [15, 16,17,18]. DOT provision for children with TB has been a challenge and deserving of additional research in the area of treatment delivery [13, 19,20,21].

As per Revised National TB Control Programme (RNTCP) guidelines in India, a DOT provider can be any person who is acceptable and accessible to the patient and accountable to the health system, except a family member [22]. This poses challenges for treating children, who may be more comfortable receiving medicines from their parents or family members than from unfamiliar DOT providers. Additionally, DOT requires healthcare resources and can be burdensome in terms of time and cost to the patient or their caretaker when attending for direct observation [7]. To the best of our knowledge, there are no studies in India that compared the treatment outcomes of children with TB based on the type of DOT provider. We conducted a non-inferiority, cluster-randomized trial in Gujarat, India, to determine if TB treatment success rates among children newly diagnosed with TB are non-inferior among those that received RNTCP-recommended DOT and those who received observed treatment from a family member.

## Methods

### Setting

Gujarat is a state in western India with a population of 60.4 million [23]. TB control services in Gujarat state have been provided by the RNTCP since 2004 and follows the WHO-recommended DOTS strategy. Currently, there are 40,897 DOT centres across 30 districts in the state. As per the RNTCP performance reports, from 2005–2011, the proportion of new paediatric cases ranged from 5 to 7% among all registered cases. In 2011, 53,110 new TB cases were registered under RNTCP; 3,219 were among children, of which, 94% were treated successfully (cured or treatment completed) [23].

The RNTCP provides a patient wise box for each diagnosed case which contains packets of loose drugs. The intensive phase consists of Isoniazid, Rifampicin, Pyrazinamide and Ethambutol to be given under direct observation thrice a week on alternate days for 2 months (24 doses). The continuation phase consists of 4 months (18 weeks; 54 doses) of Isoniazid and Rifampicin given thrice a week on alternate days, with the first dose of every weekly blister being directly observed. The box remains with the DOT provider.

Since, the numbers of tablets are too many to consume, whenever required, DOT providers are advised to crush the tablets, mix them with water, and then give to the child. It is the responsibility of the DOT provider to supervise the process of drug consumption by the child. If a child vomits within half an hour following observation, fresh dosages for all the drugs vomited should be provided by the caregiver.

### Study Design

We conducted a cluster randomized trial among children aged <15 years with newly diagnosed TB and registered for treatment under the RNTCP in Gujarat. All 30 districts in Gujarat were randomly assigned to the intervention group (n = 15) or the usual-practice group (n = 15). Eight of the 30 districts have medical colleges where approximately 60% of all childhood TB cases are diagnosed [23]. As the majority of the diagnosis of paediatric TB occurs at medical colleges, districts were randomly selected keeping equitable distribution of districts with medical colleges among the intervention and usual-practice districts. Hence, districts were first stratified based on the presence of medical colleges into two separate sets: one with a medical college and the other districts without a medical college. In both sets, the districts were alphabetically arranged and given a serial number (i.e. the first set of districts were numbered serially from 1 to 8 and in the other set numbered serially from 1 to 22). The first 4 random numbers in the first group and first 11 random numbers in the second group were allocated to intervention districts by study investigators.

### Study Population and Study Period

We calculated the sample size on the basis of the null hypothesis that treatment success rates would be similar among the intervention and usual-practice groups. Based on routinely collected program data, the TB treatment success rate was assumed to be 95% in the usual-practice arm (as well as in intervention group) and the non-inferiority limit was kept at 5% [23]. Using a sample size calculator for non-inferiority trials, with 80% power and 95% confidence, a sample size of 472 was needed [24, 25]. This was inflated by an assumed design effect of 1.2 to achieve a final sample size of 566. To achieve this sample size, we enrolled all children with newly diagnosed TB registered for treatment in Gujarat from June to September of 2012. We used standard diagnostic tools and case definitions provided in RNTCP guidelines [22]. Children who required hospitalization were excluded from the study.

### Study Procedures

In the intervention districts, study staff visited all children newly diagnosed with TB and their family members and offered the option of choosing family DOT. Family DOT was a strategy in which anti-TB medications (all the doses in intensive and continuation phase) were administered at home under the supervision of an adult household member. The adult family member was father or mother in majority of cases, but included grandparents, siblings or other relatives/guardians in rare instances. The children were enrolled after written informed consent was obtained from an adult caretaker. There were no specific criteria related to education or occupation of the family member to qualify as DOT provider. If adult caretaker or child did not want to have a family member provide DOT or there is no adult caretaker in the home, then they were considered ineligible for the intervention. In such cases an alternative mechanism (i.e., government or community DOT provider) was assigned to the child. Similar to training provided for community DOT providers (non-governmental DOT providers) the eligible family member who wished to become the DOT provider was given onsite training (at home) by government supervisory staff. The training focused on the DOTS strategy, the treatment process and its duration, the role of DOT supervisors in ensuring TB treatment completion, and side effects of anti-TB medications. The training also included component on how to manage episodes of vomiting immediate after drug consumption. In the usual-practice districts, consent was unnecessary as no alteration was made to existing treatment guidelines. The patient intake was started on 1^st^ June 2012 and last patient enrolled on 30^th^ September 2012. The Public Health System was responsible for supervising and ensuring DOT for all TB patients included in the study. The entire patient wise treatment box was provided to family member similar to a practice followed for community DOT provider. As per RNTCP guidelines, when a patient was provided treatment by a community DOT provider (non-governmental staff), there would be two treatment cards for each patient: the original treatment card with the community DOT provider and a duplicate treatment card at the health centre which was updated fortnightly by the government supervisory staff. The same system was adopted for family DOT in the intervention districts and the government-health staff updated TB treatment information. All participants were later visited by either a medical officer or a treatment supervisor assigned to their respective area. This step was taken to verify whether each child was receiving family DOT or non-family DOT according to study operating procedures. Treatment monitoring was done by following up of the children as per RNTCP guidelines (clinical/bacteriological assessment at the end of 2 months, and at the end of treatment) [22]. During the review at 2 months, a non-satisfactory response was determined by poor adherence to treatment, weight loss and worsening of symptoms. The intensive phase of treatment was extended for a month if clinical or bacteriological non-response was noted. The final treatment outcome was declared after clinical and/or bacteriological examination by the concerned medical officer of the area under whom the patient has been registered. Final treatment outcomes were defined according to RNTCP guidelines [22] The last Patient follow-up ended on 12^th^ May 2013. Before implementation, we conducted one-day training camps to familiarize RNTCP staff in all districts with the study protocol and procedures RNTCP staff then enrolled participants and assigned them to the interventions.

### Trial Registration

The authors confirm that all ongoing and related trials for this intervention are registered. Trial registration number is CTRI/2015/09/006229 The Trial Registered Retrospectively. [URL http://ctri.nic.in/Clinicaltrials/showallp.php?mid1=11438&EncHid=&userName=CTRI/2015/09/006229] The major reason for not registering this study before enrolment of participants started is that the authors believed that it is a community based trial and not a strict clinical RCT.

### Data Collection and Variables

We collected district-level health system and TB programme performance related characteristics for both the intervention and usual-practice districts. These data were extracted from the Gujarat State RNTCP programme performance reports [23]. Next, for all participants, we reviewed standard TB treatment cards kept within the peripheral health centres and abstracted information regarding TB treatment outcome, number of missed doses (as a measure of treatment adherence), type of DOT provider, age, sex, initial body weight, initial sputum smear microscopy result, site of TB disease and weight gain during treatment.

### Data Analysis

All data were double-entered into a database created with EpiData (Version 3.1, EpiData Association, Odense, Denmark). Discrepancies were corrected by going back to the source data. We performed a cluster-adjusted (taking district as the primary sampling unit) analysis using STATA (version 12.1, STATA Corp.). We compared patient-level baseline characteristics and treatment success rates (along with 95% confidence intervals) between children with TB in the intervention districts to those in the usual-practice districts using a chi-square test, Fischer’s exact test or cluster-adjusted logistic regression as applicable. The level of significance was set at ≤0.05. When comparing means, we used student’s t-test or its non-parametric alternative (Wilcoxon Mann Whitney test) depending on whether the variable was normally distributed or not (as assessed by Shapiro-Wilk test). The primary outcome was treatment success (cured and treatment completed). In our analysis, we first compared the crude (unadjusted) treatment success rates of the two DOT strategies. We then performed cluster-adjusted logistic regression and calculated adjusted treatment success rates after adjusting for potential confounding effects of age, sex and type of TB, if any. All analysis were adjusted for the clustering effect, taking district as primary sampling variable. The difference in adjusted treatment success rates between the two groups along with 95% confidence intervals around the difference was calculated. It was decided *a priori* that the new intervention would be considered non-inferior if the lower limit of the confidence interval was greater than -5%. We performed the analysis using two approaches, one intention-to-treat, and another per-protocol. The primary analysis was by intention-to-treat approach as per initial randomised allocation. The analysis was repeated (per-protocol analysis) after removal of participants who were not receiving the treatment strategies according to the study protocol (i.e., those in the usual-practice group who were receiving family DOT, and those in the intervention group receiving DOT by a non-family member). As a proxy indicator among children who successfully completed treatment, we compared treatment regularity (measured by number of missed doses) and weight gain during treatment between children in the intervention and usual-practice groups.

### Ethics Statement

Ethics approval was obtained from B J Medical College, Ahmedabad, Gujarat, and Ethics Advisory Group of the International Union Against Tuberculosis and Lung Disease, Paris, France, for the use, reporting and publication of data. The children were enrolled after written informed consent was obtained from an adult caretaker. In the usual-practice districts, consent was unnecessary as no alteration was made to existing treatment guidelines. All data were safeguarded to protect patient confidentiality and no individual patient identifiers were retained.

## Results

**Fig 1:**
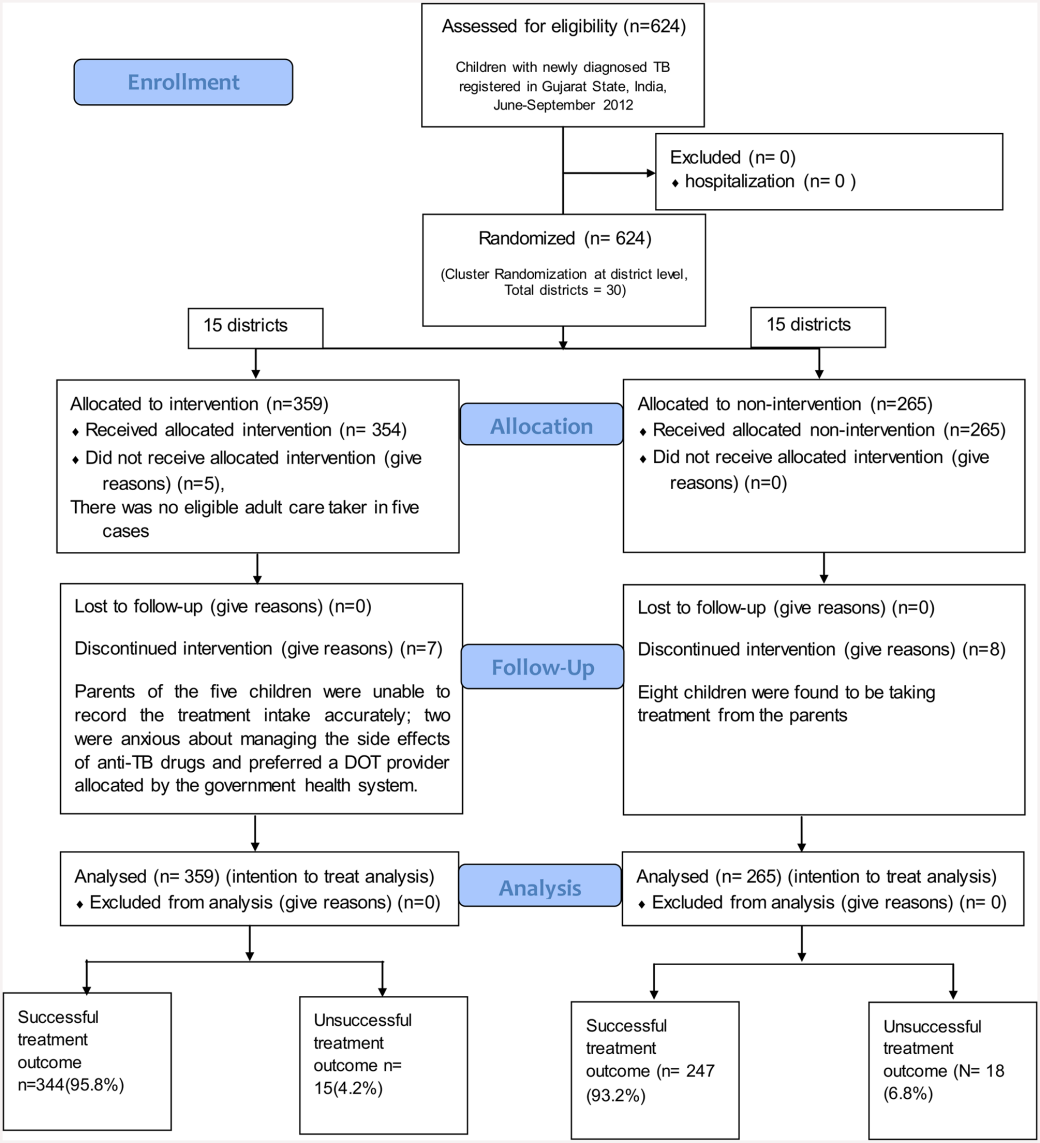
Flow diagram of study cohort of children with newly diagnosed TB registered in Gujarat State, India during June-September 2012.

District-level health system, and TB programme related characteristics for the intervention and usual-practice districts are shown in Table 1 [23]. The intervention and non-intervention districts were similar to each other on a host of health system related and TB performance indicators including baseline treatment outcomes among paediatric TB patients. A total of 624 children with newly diagnosed TB were registered during the study period: 359 in the intervention districts and 265 in the usual-practice districts under RNTCP. None of the children required hospitalization; thus, all were included in the study. The CONSORT flow diagram on enrolment, allocation, follow-up and analysis is shown in Fig 1.

**Table 1:** Baseline characteristics of districts by intervention, year 2011 [23].

Variable	Family DOT (Intervention districts)	Non-Family DOT (Usual-practice districts)	P value (Chi-square test)
Population (n)	33,165,080	25,854,920	
**Health system infrastructure**			
TB Unit^a^ (n)	81	63	
Peripheral Health Institutions (n)	1058	882	
Designated Microscopic Centre (n)	440	308	
Medical College (n)	8	6	
Medical officer TB Unit	81	63	
Senior Treatment Supervisor	81	63	
**TB program performance**			
Proportion of child TB cases of new cases	5.9%	5.2%	<0.001
Proportion of smear-positive TB patients started on treatment within seven days of diagnosis	92.4%	92.4%	0.94
Proportion of smear-positive TB patients registered within a month of starting treatment	98.3%	97.5%	0.54
Treatment success rate of all new smear positive TB patients (%)	87.5%	88.1%	0.68
**Treatment outcomes** of children^b^	2120	1099	
Cured	333 (16%)	204 (18%)	0.37
Treatment completed	1664 (79%)	855 (78%)	
Death	55 (3%)	26 (2%)	
Failure	12 (1%)	4 (0.3%)	
Defaulted	41 (2%)	15 (1%)	
Transferred out	8 (0.3%)	4 (0.3%)	

^a^ Tuberculosis Units [TU] (geographical areas defined as sub-district level programme management units, each covering a population of 250000–500000 with TB diagnostic and treatment services being delivered through a network of primary, secondary and tertiary healthcare facilities)

^b^ aged <15 years with newly diagnosed TB and registered for treatment under the RNTCP in Gujarat. Fischer’s exact test was used since several cells had expected value less than 5.

### Intention to Treat Analysis

Baseline characteristics among children newly diagnosed with TB in the intervention and usual-practice groups were similar with respect to age, sex, pre-treatment sputum smear results, type of TB, and pre-treatment body weight (Table 2). Thirty (4.8%) paediatric TB patients were aged below 1 year and 190 (30%) were below 5 years. Treatment success rates were 95.8% (95% CI: 94.1%–97.1%) and 93.2% (95% CI: 88.9%–95.9%) (p = 0.11) among the intervention and usual-practice districts respectively. The respective adjusted treatment success rates were 95.9% (95% CI: 94.7%–97.2%) and 92.9% (95% CI: 89.9%–96.0%). The difference in treatment success rates between the two groups was 3.0% (95% CI: -0.7%–+6.7%). Since the lower limit of the 95% confidence interval (-0.7%) is greater than -5% (the non-inferiority limit), the new intervention was concluded to be non-inferior to the existing intervention. The actual design effect for the key outcome variable was found to be 1.02 (less than the assumed 1.2 in sample size calculations).

**Table 2:** Comparison of baseline characteristics among children with newly diagnosed TB, by treatment strategy, registered in Gujarat State, India, June-September 2012.

Characteristics	Treatment Strategy				P-value
	Family DOT (Intervention district)		Non-Family DOT (Usual-practice districts)		
	Number	%	Number	%	
**Total**	359	100	265	100	
**Sex**					
Male	163	45.4	135	50.9	0.17
Female	196	54.6	130	49.1	
**Age in years**^a^					
0 to 1 year	20	5.6	10	3.8	0.57
2 to 5 years	90	25.3	70	26.4	
6 to10 years	124	34.8	85	32.1	
11 to 14 years	122	34.3	100	37.7	
**Disease Classification**					
New smear positive pulmonary TB	58	16.2	43	16.2	0.14
New smear negative pulmonary TB	28	7.8	34	12.8	
New extrapulmonary TB	196	54.6	143	54.0	
New Other^b^	77	21.4	45	17.0	
**Initial Weight (in Kg)**^c^					
Median (IQR)	17	(11)	17	(13)	0.54

Kg = Kilogram, TB = Tuberculosis, DOT = Directly Observed Treatment, SD = Standard Deviation, Treatment success = Cured or treatment Completed.

Note: Chi-square test was used for assessing difference between the proportions.

^a^ Age was not recorded for 3 patients.

^b^ A patient who does not fit into the any of the types mentioned above (smear positive, smear negative, extra pulmonary), where bacteriological evidence could not be demonstrated but decision to treat was taken on clinical grounds would continue to be recorded and reported as “OTHERS”.

^c^ Initial weight was not recorded for 1 person; Wilcoxon Mann Whitney test (a non-parametric test) was used for assessing statistical significance since the variable was not normally distributed.

The difference between treatment outcomes disaggregated by the type of TB is shown in Table 3. It may be noted that the intervention was either non-inferior or superior to the usual-practice group across the types of TB patients. Out of the total 101 new smear positive cases (58 in intervention group and 43 among non-intervention group) 85 had converted to smear negative at the end of two months (50 (86.2%) and 35 (81.4%) in the two groups respectively) with 4 (6.9%) and 3 (7%) non-converters respectively (p = 0.23). At the end of treatment, 47 (81.0%) in the intervention group and 35 (81.4%) in the non-intervention group were bacteriologically cured (p = 0.22). Although default was a rare event (n = 14), when compared to treatment success, risk of default was higher in the usual-practice districts as compared to intervention districts (p = 0.03).

**Table 3:** Comparison of treatment outcomes among children with newly diagnosed TB, stratified by disease classification and treatment strategy, registered in Gujarat State, India, June-September 2012.

Characteristics	Treatment Strategy				P-value
	Family DOT (Intervention district)		Non-Family DOT (Usual-practice districts)		
	Number	%	Number	%	
**All TB patients**	359	100	265	100	
Cured	47	13.1	35	13.2	0.17
Treatment completed	297	82.7	212	80.0	
Death	5	1.4	6	2.3	
Default	4	1.1	10	3.8	
Transfer Out	3	0.8	2	0.8	
Failure	3	0.8	0	0.0	
**New smear-positive PTB**	58	100	43	100	
Cured	47	81.0	35	81.4	0.22
Treatment completed	2	3.4	4	9.3	
Death	3	5.2	3	7.0	
Default	3	5.2	0	0.0	
Transfer Out	0	0.0	1	2.3	
Failure	3	5.2	0	0.0	
**New smear-negative PTB**	28	100	34	100	
Treatment completed	28	100.0	33	97.1	1.0
Death	0	0.0	1	2.9	
Default	0	0.0	0	0.0	
Transfer Out	0	0.0	0	0.0	
Failure	0	0.0	0	0.0	
**New others** ^a^	77	100	45	100	
Treatment completed	76	98.7	42	93.3	0.05
Death	1	1.3	0	0.0	
Default	0	0.0	3	6.7	
Transfer Out	0	0.0	0	0.0	
Failure	0	0.0	0	0.0	
**New extra-pulmonary TB**	196	100	143	100	
Treatment completed	191	97.5	133	93.0	0.02
Death	1	0.5	2	1.4	
Default	1	0.5	7	4.9	
Transfer Out	3	1.5	1	0.7	
Failure	0	0.0	0	0.0	

^a^ A patient who does not fit into the any of the types mentioned above (smear positive, smear negative, extra pulmonary), where bacteriological evidence could not be demonstrated but decision to treat was taken on clinical grounds would continue to be recorded and reported as “OTHERS”.

Note: Fischer’s exact test was used for assessing difference between the proportions as the expected cell values is many of the cells were less than 5.

### Per Protocol Analysis

Of 359 children in the intervention districts, 12 did not receive family DOT. Five of these children did not have an eligible family member who could deliver DOT, and parents of the remaining seven children were unable to record the treatment intake accurately or were anxious about managing the side effects of anti-TB drugs and preferred a DOT provider allocated by the government health system. A Government DOT provider assumed DOT responsibilities in these cases. Therefore, a total of 347 children received family DOT, an acceptance rate of 97%. Of the 347 who received family-DOT, 189 received DOT from their father, 118 from their mother, 7 from grandparents, 14 from siblings, and 19 from others. Contrary to guidelines, eight (3%) of 265 children were receiving DOT by a family member in the usual-practice districts. Among the remaining patients, 103 received DOT from a government health care worker, 151 from community DOT providers, and 3 did not have the type of DOT provider recorded.

The analysis was repeated after excluding the 20 children who were not receiving treatment according to the initial study allocation (per protocol analysis). The adjusted treatment success rates among the intervention group (n = 347) and usual-practice group (n = 257) were 95.8% (95% CI: 94.6%-97.0%) and 92.7% (95% CI: 89.6%-95.8%) (p = 0.10), respectively, with a difference between the two groups of 3.1% (95% CI: -0.6%-6.9%).

Our comparison of treatment regularity and weight gain during treatment, by treatment strategy are presented in Table 4. There were no statistically significant differences in these characteristics between the two groups.

**Table 4:** Comparison of individual level factors among children successfully treated for TB, by treatment strategy, registered in Gujarat State, India, June-September 2012.

Characteristics	Treatment Strategy				P-value
	Family DOT (Intervention districts)		Non-Family DOT (Usual-practice districts)		
	Number	%	Number	%	
**Total (treatment success)**	344	100	247	100	
**Number of Missed doses during treatment**^a^					
None	271	78.8	181	73.9	0.19
1–3 doses	47	13.7	35	14.3	
≥4 doses	26	7.6	29	11.8	
**Weight gain during treatment (in Kg)**					
Mean (SD)^b^	2.86	(2.30)	2.67	(1.69)	0.88

^a^ Treatment card was missing information on dosage information for 2 children. Chi-square test was used for assessing difference between the proportions.

^b^ Weight gain could not be determined for 46 children whose end of treatment information weight was missing. We used Wilcoxon Mann Whitney test (a non-parametric test) for assessing statistical significance since the variable was not normally distributed.

Since the treatment success rates were high across subgroups of exposure variables (like sex, type of TB, age groups) with little differences from a clinical or programmatic perspective and unavailability of sufficient numbers across the sub-groups, we refrained from exploring effect modification or interaction.

## Discussion

We observed a high acceptance rate (97%) for family DOT. Family DOT achieved similar treatment success rates to that of non-family member DOT among children with TB in Gujarat. The intervention and the usual-practice groups were similar to each other by age, sex, type of TB and baseline weight. This indicates the success of randomization and that the groups were comparable to each other with the difference, or lack of, in outcomes being more likely to be attributable to the intervention. Both strategies achieved the desired WHO target for treatment success under programmatic conditions [26]. These results support those obtained in earlier studies among patients of all age groups which showed that family-member and community DOTS strategies can achieve desired success rates [27–29]. Since the study was conducted under routine programme conditions, these results may be generalizable to other similar settings.

The high level of treatment adherence recorded in the intervention group indicates good uptake of the intervention among family members. Satisfactory weight gain was observed during treatment in both groups, which is an indirect indicator for response to anti-TB treatment [30]. Although family DOT was widely accepted, there were situations in which there was no adult family member available or qualified to provide DOT, and situations when parents preferred a health system allocated DOT provider. Therefore, it is important for TB programmes to maintain capacity to provide DOT providers while allowing families the option to provide DOT for their children. Although we found no statistical difference in treatment outcomes according to treatment strategy, a total of 11 deaths (5 from the family-DOT group and 6 from the usual practice group) were observed among this cohort of paediatric patients. This is concerning and worth noting. Unfortunately, we are unable to comment on the exact causes of death, or to assess potential causes of death with data collected for this study.

One argument against the inclusion of a family member as a DOT provider is that ***“****cultures with strong matriarchal or patriarchal structures*, *it is not realistic to believe that any member of the family can insist on any behaviour by the dominant family head”* [15]. While this may not be true even in adults with TB, the same certainly not apply to children who, given the cultural context in India, are likely to follow instructions and encouragement by their parents or guardians. A Cochrane review suggested that DOT ***“****is expensive to implement*, *and there appears to be no sound reason to advocate its routine use until we better understand the situations in which it may be beneficial*” [9]. The family-member DOT strategy has the advantage over standard DOT in that it does not involve community volunteers such as ASHA (Accredited Social Health Activist) or Anganawadi workers, who are becoming increasingly overloaded because of the many different health programmes requesting their help.

### Study Strengths

This study was the first to assess the effectiveness of family-based DOT conducted in children in India. It was performed in routine program settings and hence provides evidence for wider scale-up. Randomization was successful and resulted in two comparable groups enabling a clean assessment of the effect of the intervention. The study was conducted across the entire state of Gujarat ensuring a large representative sample. There were very few children who did not adhere to the assigned treatment method outlined in the protocol (e.g., family-based vs. non-family-based DOT), and even after the removal of these children there was no difference in the findings. Finally, this study demonstrated that family members can be easily trained to provide DOT for children which can potentially lead to a more affordable strategy allowing for shifting of resources to accelerate other TB program activities.

### Limitations of the Study

There were some limitations. First, the study was performed in a programmatic setting and used programmatic definitions for treatment outcomes, in which, the majority of paediatric TB patient’s outcome was not verified microbiologically. This is an acknowledged, general challenge in children with TB given the difficulties in collecting good quality sputum specimen for microbiologic examination. This is even more challenging at the end of the treatment when most patients are symptom-free and cannot produce sputum. Second, the study does not have data on long term follow-up, such as relapse rates for study participants, which should be a topic for future research. We intend to follow-up this cohort for a period of one year to assess the relapse rates and report on this separately. Because of the study design, no blinding could be performed. Third, while the two groups were similar with reference to age and baseline weight, we did not have any information with respect to height. Hence, we will not be able provide information on the proportion of failure to thrive or any other measure of malnutrition at baseline. Given the potential of this variable to act as a possible confounder, we acknowledge this missing information a limitation. The same limitation is applied for other comorbid situations such as diabetes and HIV. Fourth, presence of other TB patients in the household and whether they functioned as treatment observers for the study group was not systematically captured. Hence we are not able to comment if the two groups were different with respect to this variable and adjust for the same in analysis. Fifth, we could not perform a drug susceptibility test among children who failed treatment and hence do not have any information on acquired drug resistance. Finally, side effects from anti-TB medications were not documented completely and hence we cannot assess if differential rates of such side effects occurred among the two groups.

### Implications and Recommendations

Given the resource limitations in India, a shift toward family-based DOT for children with TB could decrease the work load of an overburdened health system and reduce the cost of treatment of childhood TB. Based on results of this study, India has revised national guidelines to allow the option of having a family member provide DOT for children with TB [31, 32].

## Conclusion

DOT by a family member is not inferior to that provided by a non-family member among new TB cases in children and can attain international targets for treatment success under programme conditions.

## Supporting Information

S1 CONSORT Checklist (DOC)

S1 Dataset Data files. (ZIP)

S1 Format Data Entry Format. (ZIP)

S1 Protocol Study Protocol. (ZIP)
