# Supplementary material for: Direct Observation of Treatment Provided by a Family Member as Compared to Non-Family Member among Children with New Tuberculosis: A Pragmatic, Non-Inferiority, Cluster-Randomized Trial in Gujarat, India
Source: PLoS One. 2016 Feb 5;11(2):e0148488. doi: 10.1371/journal.pone.0148488 (PMC4743945; doi:10.1371/journal.pone.0148488)
Supplement: S1 Protocol — (ZIP) [file pone.0148488.s004.zip › approved pediatric or protocol/01 Annexure 5 Consent form.doc]

	Family DOT study
Consent form	


Project title: Prospective study on inclusion of Family member as a DOT provider for Child TB Patient in State of Gujarat	

  Name of Principal investigator	Dr Paresh Dave	
Address of principal investigator	Department of Health & Family Welfare, Dr. Jivaraj Mehta Bhavan, Block-5, Government of Gujarat, Gandhinagar, Phone-079-23253330.	
Email address of principal investigator	stogu@rntcp.org	
Please read the information below and then write your initials in the grey boxes.
1.	I confirm that I have read and understood the information sheet dated ____/	/___ for the above study and that I have had a chance to ask questions.
2.	I confirm that I have had enough time to think about whether or not I want my child to be included in the study. 
3.	I confirm that I am happy for researchers in the Study team to have access to the results of my child's routine follow up. 
4.	I understand that I do not have to allow my child to take part in the study and I can withdraw my child from the study at any time, without giving any reason, without my child's medical care or legal rights being affected. 
5.	I agree that if I choose to withdraw my child from the study I give study researchers permission to keep and use data before this withdrawal. I understand that I can withdraw this permission at any time. 
6.	I understand that researchers involved in the study at this Peripheral Health Institutions_________________	(PHI name) or regulatory authorities whenever required may call my child for physical examination and look at the Treatment notes and the records of my child. This will allow them to check that the study has been run correctly. I give permission for these individuals to have access to these records. 
7.	I agree to allow my child to take part in the Family DOT study. 
8.	I agree that Mr/Ms_____________ will be a DOT provider for my child's treatment    
9.	I agree to allow small samples of my child's sputum to be retained for up to 3 Months and then discarded after analysis. 
10.	I agree that the study team may contact me in the future regarding this study. 
Name of child patient:	Birth date:	/	/		
			
Name of adult family member 
(DOT provider )	Signature 	Date ___/____/____	
			
Independent witness	Witness signature	Date___/____/____	
			
Name of the Parents or Guardian	Signature	Date___/____/____	
			
Investigator (to be contacted
if there are any problems)	Signature	Date___/____/____		
