# Supplementary material for: Direct Observation of Treatment Provided by a Family Member as Compared to Non-Family Member among Children with New Tuberculosis: A Pragmatic, Non-Inferiority, Cluster-Randomized Trial in Gujarat, India
Source: PLoS One. 2016 Feb 5;11(2):e0148488. doi: 10.1371/journal.pone.0148488 (PMC4743945; doi:10.1371/journal.pone.0148488)
Supplement: S1 Protocol — (ZIP) [file pone.0148488.s004.zip › approved pediatric or protocol/001 Protocol Family DOT services for children with TB Gujarat.doc]

**Prospective study on inclusion of the family member as a DOT provider for children with TB in state of Gujarat**

**Investigators**

Paresh Dave^1^, Amar Shah^2^, Pankaj Nimavat^1^, Ajay MV Kumar^3^, Kirit Pujara^1^, Pradip Patel^1^, K Maheria^1^

1. Department of Health, Government of Gujarat, India
2. WHO country office in India, New Delhi, India.
3. International Union against Tuberculosis & Lung Disease, South-East Asia Regional Office, New Delhi, India.

**Background**

Directly observed therapy (DOT) is a supportive mechanism where a tuberculosis (TB) controller directly observes a patient taking their medication to ensure treatment adherence. As per the Revised National Tuberculosis Control Programme (RNTCP) guidelines, A DOT provider is a person who administers DOT and is either a community or government health worker and not a family member. In Gujarat as per the RNTCP reports from 2005 to 2011, the proportion of new paediatric cases ranges from 5 to 7% among all registered cases. An average treatment success rate among paediatric patients is 94%. (As per RNTCP, the paediatric age group is child between 0-14 years)

**Rationale**

As per feedback received by district TB officials and found during internal evaluations, DOT provision for children with TB is a challenge. It is generally assumed that children are more comfortable taking DOT from their parents/family members rather than from unfamiliar DOT providers. During supervisory visits it was found that, against RNTCP guidelines, DOT is often provided by a family member. A cluster randomized controlled trial among adult new smear positive pulmonary TB patients in Nepal showed that the family-member DOTS and community DOTS strategies can both attain international targets for treatment success under programme conditions, and both strategies might also be appropriate in other parts of the world where directly observed treatment by health workers is not feasible^[1]^. Another study concluded that *“DOT at both community and family levels achieves high clinical outcome (over 80%) at relatively low costs per successful treatment and both strategies or variants there of are worthy of consideration by NTPs contemplating moving towards more patient- friendly supervision and patient support as recommended in the new Stop TB strategy and the ISTC.”* ^[2]^ This study will attempt to document treatment adherence and treatment outcomes among children with TB that received DOT from a family member and compare those to children with TB who received DOT from a non-family member.

**Research question**

Does a *Family Member* providing DOT leads to adherence and treatment outcomes similar to a *Non-Family Member* providing DOT among new TB paediatric patients registered in months of June-September 2012 under RNTCP in the state of Gujarat?

**Objective of the Study**

1. To determine the difference in TB treatment success rates among children diagnosed with TB that received WHO-recommended DOT and those who received observed therapy from a family member registered in Gujarat State, June-September 2012.
2. To compare treatment regularity, weight gain during treatment, and need for extension of the intensive phase of treatment among children successfully treated for TB, by treatment strategy.

**Methodology**

**Study Design:** Prospective cohort study

**Setting**

Gujarat, with a population of about 60 million and consisting of 30 districts and 138 programme management units (TB units), has been chosen to conduct the evaluation. There are 8 districts, having government medical colleges which contribute the majority of diagnosis of child TB cases. As per the RNTCP reports, from 2005 to 2011 the proportion of new paediatric cases ranges from 5% to 7% among all registered cases. In 2011, a total of 53110 new cases were registered under RNTCP in the state and among them 3219 cases were new paediatric cases. Contribution of NEP, NSN and NSP is 50%, 30% and 20% respectively. The average success rate among NEP, NSN and NSP is 95%, 95% and 91% respectively.

**Study Population and Study Period**

The study will include new paediatric TB patients registered under RNTCP in Gujarat. The sample size is calculated using **non-inferiority trial^[3]^** as below.

| Two-sided significance level(1-alpha): | 95 |
| --- | --- |
| Power(1-beta, % chance of detecting): | 80 |
| Ratio of sample size, Unexposed/Exposed: | 1:1 |
| Percentage 'success' in standard treatment group | 95 |
| Percentage 'success' in intervention group | 95 |
| Non-inferiority limit, **d** | 5 |
| Sample size required per group | 236 |
| Total Sample size | **472** |
| Final sample size (considering 1.2 design effect) | **566** |

Considering the sample size of around 600, paediatric cases registered in months of June-Sept 2012 will constitute the study population.

**Protocol Activity Flow Chart**

Ethical approval from the concern Authorities

Districts Randomized (with medical college equal distribution stratification)

**Allocation**

15 Districts allocated strictly Non- Family DOTs

15 Districts Allocated to the option of Family DOTS

**Training (Training on Annexure: 1, 2, 3, 4, 5, 6)**

| Category of staff | Training by whom | Time period |
| --- | --- | --- |
| All District TB officers and key RNTCP contractual staff | Study investigators | 1^st^ week May 2012 |
| Medical officers PHI | District TB officers | May 2012 (during monthly PHI meeting) |
| Medical College faculties | District TB officers | May 2012 |

**Enrolment**

All Paediatric TB new cases registered under RNTCP in month of June-September 2012 will be provided option for choosing family DOTS.

All Paediatric TB new cases registered under RNTCP in month of June-September 2012 will be under the RNTCP current system

On the spot training will be imparted for the DOT provider, by the Peripheral Health Worker (PHW), regarding directly observed treatment, adverse reactions, follow-up sputum examination and recording of drug intake, before starting treatment. The PHW (Peripheral Health Worker) is responsible for supervising and ensuring DOT and updating of the original treatment card at the PHI on a fortnightly basis. All study participants will be interviewed by MOTC or STS of their respective TB Unit and they will fill up the supervisory Annex-2 at least monthly during Intensive Phase of treatment. This step is crucial to determine whether paediatric patient is receiving Family DOT or Non-Family DOT.

**Follow-up**

Follow-up of children with TB will done as per RNTCP guideline. Clinical/bacteriological assessment will be done at end of 2 months and at the end of treatment. This will be noted in the Annexure-I

**Data Collection**

Annexure-1, 2 and 5,6 will be collected by end of 2 months treatment. Copy of treatment card, will be collected at the end of intensive phase of treatment and at the end of treatment. Data Documentation sheet will be filled up from treatment card and entered in to electronically using Epi-data software.

**Procedure (as explained in flow chart)**

The state will be divided in to two subgroups: 15 districts will receive the intervention and 15 districts will operate under RNTCP guidelines. Districts will be randomly selected keeping equitable distribution of medical colleges among interventional and non interventional districts (using Open Epi).

Before implementation in all districts, 1 day training for- staff will be undertaken. The training will include following topics

- Study protocol
- Study formats and data needed to be collected (Annexure 1 to 6)

In the interventional districts adult family member will be given the choice to become the DOT provider. If the family member/patient doesn’t wish to be a DOT provider or if no eligible DOT provider can be identified, an alternative mechanism (i.e. Government or Community DOT provider) will be assigned to the patient in consensus with the patient. Child will receive the same TB treatment from either Government or Community DOT provider as the rest of the patients. Doctor of concerned PHI, Health worker and trained community DOT provider of your area together with the whole team, will closely take care of your child during treatment help you to give advice to overcome these side effects.

The eligible family member who wishes to become the DOT provider will be provided ‘RNTCP DOT provider’ on the job training for 1 day, which focuses on the DOTS strategy, the treatment process and its duration, the role of DOT supervisors in ensuring tuberculosis treatment completion, the side effects of tuberculosis drugs, and the process of referring patients for their management. The same will be undertaken for each and every eligible DOT provider in the non intervention districts as per RNTCP protocol. The PHW (Peripheral Health Worker) is responsible for supervising and ensuring DOT and updating of the original treatment card at the PHI on a fortnightly basis. All participants will be determined by MOTC or STS of their respective TB Unit and filled up the supervisory Annex-2 at least once during Intensive Phase of treatment. This step is crucial to determine whether paediatric patient is receiving Family DOT or Non-Family DOT. Follow-up of children with TB will be done as per RNTCP guideline (Clinical/bacteriological assessment at end of 2 months, and at the end of treatment). This will be noted in Annexure-I.

Annexure-I, II and 5,6 will be collected by end of 2 months treatment. Copy of treatment card, will be collected at the end of intensive phase of treatment and at the end of treatment. Data Documentation sheet will be filled up from treatment card and entered in to electronically using Epi-data software.

**Study Duration**

The study participant’s enrolment will be for 3 months and total duration will be for 12 months in which their treatment outcome will be measured.

**Inclusion and Exclusion criteria**

**Inclusion Criteria:** Child age between (0 to 14) with TB registered between June 2012 to September 2012 who gives assent (if applicable) and his/her parents gives consent to participate in the study will be included in the study.

**Exclusion Criteria:** In following situation child suffering from TB will be excluded from the study.

1. Very sick children referred for hospital treatment.
2. If parents are not available.
3. If parents/child do not give consent/assent

**Study Definition**

- **Family DOTS:** A strategy with drug taking supervised by any adult household member of paediatric TB Patients who has volunteered to administer DOT as per the programme guidelines, and who is willing to undergo on-job training on DOT provision.
- **Missed dose:** When a TB patient scheduled to take a dose of anti-TB medicines does not show up to a DOT centre and misses that particular dose on that day. Single missed dose can be taken on the next day on successful retrieval of the patient before the next dose and will not amount to treatment interruption.
- **Treatment interruption:** When a missed dose is not taken by the patient on the subsequent day or days thus leading to non administration of anti-TB doses for more than 1 consecutive day or leading to postponement of at least one dose will amount to a treatment interruption.
- **Final Treatment Outcome:** Final treatment outcome will be written by the respective Medical Officer, PHI in the final outcome row of the treatment card.
  a. The paediatric TB Patient who has taken the full course of treatment under RNTCP and getting final treatment outcome as *cured* or *Treatment Completed* will considered as favourable final treatment outcome
  b. Any paediatric TB patient not fulfilling the above criteria will be given unfavourable treatment outcome (died, default or failure)

**Data collection procedures and Source of data:**

A copy of the treatment card will be collected at the end of the intensive phase of treatment and at the end of treatment. A data documentation sheet will be filled out from the treatment card and entered electronically using Epi-data software and will be maintained at the State TB Training & Demonstration Centre, Ahmedabad.

Sources of data

i. Treatment card
ii. TB register
iii. Developed formats (Annexure:1,2)

**Variables :**

**Dependent/outcome Variables**

- Treatment regularity
- Final Treatment Outcome

**Independent Variables**

- Type of DOT provider
- Age of the paediatric patient
- Sex of patient
- Initial weight before starting treatment
- Weight after 2 months of the treatment
- Initial sputum result
- Follow up sputum result
- Extension of IP
- Total duration for completing IP phase of treatment
- Missed doses in IP
- Consistency between Blisters and treatment card
- Interruption of treatment in IP
- Site of TB
- Cadre of the DOT provider (in case, Non family person is a DOT provider)
- Relation to the patient (in case, family member is a DOT provider)
- Total duration for completing IP phase of treatment
- Actual DOT provider (will find from supervisory visit by staff)

**Data entry:**

A copy of treatment cards will be collected at the end of intensive phase of treatment and at the end of treatment. A Data Documentation sheet (annexure: 3) will be filled out from the treatment card by the study team. This then entered electronically using EpiData software at the state level by an RNTCP data entry operator. Double data entry will be done by two DEO independently. Both databases will be compared. All discrepancy in the data entry will be resolved through referral to original data collection format and treatment cards. Finalized database will be securely locked for safety.

**Ethical Considerations**

The study will be conducted after ethical approval from Advisory Group of The Union and local ethics committee of B J Medical College, Ahmedabad.

**Consent and confidentiality**

Patients enrolled are already identified as paediatric TB patients in the RNTCP programme records. Data would be maintained securely by programme staff, and electronic databases would contain no personal identifiers.

In the interventional district where, all paediatric newly diagnosed cases will be offered a Family DOT, an Informed consent would be taken from paediatric patients and parents or guardians (Annexure 4, 5, 6). No monitory support or incentive will be provided to the family or the patient for accepting the Family DOT.

All data collected for the study will be kept confidential and will be accessible to only the study investigators and the field staff of the programme. While disseminating the study findings to the external audience, only pooled/aggregate results will be shared. No references will be made to any individual patient/staff.

**Impact and value to TB control activities**

The comparative analysis on the treatment adherence and treatment outcome among paediatric patients will suggest on decision on the inclusion of a family member as a DOT provider for paediatric patients.

**P**lan of activities

| **Activity** | Apr 12 | May 12 | Jun 12 | Jul 12 | Aug 12 | Sep 12 | Oct 12 | Nov 12 | Dec 12 | Jan  13 | Feb 13 | Mar 13 | April 13 | May 13 |
| --- | --- | --- | --- | --- | --- | --- | --- | --- | --- | --- | --- | --- | --- | --- |
| Submission of protocol | ✓ |  |  |  |  |  |  |  |  |  |  |  |  |  |
| Ethical clearance | ✓ |  |  |  |  |  |  |  |  |  |  |  |  |  |
| Administrative approval | ✓ |  |  |  |  |  |  |  |  |  |  |  |  |  |
| Training of Staff | ✓ | ✓ |  |  |  |  |  |  |  |  |  |  |  |  |
| Recruitment |  |  | ✓ | ✓ | ✓ |  |  |  |  |  |  |  |  |  |
| Purchase of logistics | ✓ |  |  |  |  |  |  |  |  |  |  |  |  |  |
| Finalization of manuals | ✓ | ✓ |  |  |  |  |  |  |  |  |  |  |  |  |
| Data collection |  |  | ✓ | ✓ | ✓ | ✓ | ✓ | ✓ | ✓ | ✓ | ✓ | ✓ |  |  |
| Coding of data |  |  |  |  | ✓ | ✓ | ✓ | ✓ | ✓ | ✓ | ✓ | ✓ |  |  |
| Data entry |  |  |  |  |  |  |  |  |  | ✓ | ✓ | ✓ | ✓ |  |
| Data analysis |  |  |  |  |  |  |  |  |  | ✓ | ✓ | ✓ | ✓ |  |
| Interpretation of results |  |  |  |  |  |  |  |  |  |  |  | ✓ | ✓ | ✓ |
| Submission of final report |  |  |  |  |  |  |  |  |  |  |  |  | ✓ | ✓ |

1. James N Newell et al. Family-member DOTS and community DOTS for tuberculosis control in Nepal: cluster-randomised controlled trial Lancet 2006; 367: 903–09
2. Community-based DOTS and family member DOTS for TB control in Nepal: costs and cost-effectiveness. Tolib Mirzov et al. Bio med central *Cost Effectiveness and Resource Allocation* 2008, 6:20 Available at http://www.resource-allocation.com/content/6/1/20
3. Blackwelder WC. "Proving the Null Hypothesis" in Clinical Trials. *Control. Clin. Trials* 1982; **3**:345-353
