# Supplementary material for: Direct Observation of Treatment Provided by a Family Member as Compared to Non-Family Member among Children with New Tuberculosis: A Pragmatic, Non-Inferiority, Cluster-Randomized Trial in Gujarat, India
Source: PLoS One. 2016 Feb 5;11(2):e0148488. doi: 10.1371/journal.pone.0148488 (PMC4743945; doi:10.1371/journal.pone.0148488)
Supplement: S1 Protocol — (ZIP) [file pone.0148488.s004.zip › approved pediatric or protocol/01 Annexure 1.doc]

**Annexure: 1**

Clinical Assessment guideline

(This RNTCP algorithm should be used by Doctor during clinical assessment after 2 month)

Final Assessment: 🞏 Satisfactory response 🞏 Non-Satisfactory response
