# Supplementary material for: Direct Observation of Treatment Provided by a Family Member as Compared to Non-Family Member among Children with New Tuberculosis: A Pragmatic, Non-Inferiority, Cluster-Randomized Trial in Gujarat, India
Source: PLoS One. 2016 Feb 5;11(2):e0148488. doi: 10.1371/journal.pone.0148488 (PMC4743945; doi:10.1371/journal.pone.0148488)
Supplement: S1 Protocol — (ZIP) [file pone.0148488.s004.zip › approved pediatric or protocol/01 Annexure 2.doc]

**Annexure: 2**

Supervisory visit form by MO/BHO/STS (for all registered new pediatric Patients from June-September 2012)

(This form should be filled up by MO/BHO/ STS during home visit of a patient and to concern DOT provider)

Name of District: **___________**

Name of Patient:

Age:	Sex:	Weight:

Village:

PHC:-

Block:

Name of TU:

TB No:

Designation of HCW: tick one. MO/ BHO/STS

Date of Visit:	/	/ 20

DOT Provider:  Family Member  Non Family Member

No. of Vacant Blisters in the PWB on the day of visit:

No. of Filled Blisters in the PWB on the day of visit:

No. of Tick Mark in the Treatment Card: *(Please write down number of tick mark in given blanks)*

√……………. √…............ ..........
