# Supplementary material for: Direct Observation of Treatment Provided by a Family Member as Compared to Non-Family Member among Children with New Tuberculosis: A Pragmatic, Non-Inferiority, Cluster-Randomized Trial in Gujarat, India
Source: PLoS One. 2016 Feb 5;11(2):e0148488. doi: 10.1371/journal.pone.0148488 (PMC4743945; doi:10.1371/journal.pone.0148488)
Supplement: S1 Protocol — (ZIP) [file pone.0148488.s004.zip › approved pediatric or protocol/01 Annexure 3 Data collection word.doc]

**Annexure: 3**

**(Data Documentation Sheet: derived from treatment card: Secondary Data)**

1. District:
2. TB Unit:
3. TB number:
4. Age:
5. Sex:
6. Which card was reviewed: 🞏Original 🞏Duplicate
7. Initial home visit made by: 🞏 GHS staff 🞏 STS🞏 TBHV🞏 other (specify)
8. DOT Provider: 🞏 Family Member 🞏 Non Family Member
9. If Non family member: Designation of DOT Provider:_____________
10. If Family Member: relation to the patient:___________
11. Classification of TB:________
12. No of missed doses in IP …………… of these number of occasions retrieval action taken:……
13. No. of Interruption during treatment……………
14. Initial Sputum result:
15. Follow-up Sputum result:
16. Initial weight before starting treatment:
17. Weight after 2 months of treatment:
18. Extension of IP: Yes / No
19. If extension of IP doses taken in 3 months____
20. Total duration of IP in days:……….
21. Interim outcome of Treatment:- favourable/ unfavourable
22. Total duration of treatment in days:……….
23. Final Treatment Outcome: Cured/Treatment completed/Failure/ Defaulted/ Transferred Out
24. Consistency between treatment card and PWB check during supervisory visit: yes/no
25. Actual DOT provider (will find from supervisory visit either by Senior Treatment Supervisor or Medical Officer- Tuberculosis Unit)
