# Supplementary material for: Direct Observation of Treatment Provided by a Family Member as Compared to Non-Family Member among Children with New Tuberculosis: A Pragmatic, Non-Inferiority, Cluster-Randomized Trial in Gujarat, India
Source: PLoS One. 2016 Feb 5;11(2):e0148488. doi: 10.1371/journal.pone.0148488 (PMC4743945; doi:10.1371/journal.pone.0148488)
Supplement: S1 Protocol — (ZIP) [file pone.0148488.s004.zip › approved pediatric or protocol/01 Annexure 4 information sheet.doc]

**Annexure: 4**

# Parent/Guardian/Patient information sheet

Dear Madame/Sir:

**Prospective study on inclusion of Family member as a DOT provider for children with TB in state of Gujarat**

Thank you for reading this information sheet

**Why Directly Observed Treatment needed for TB treatment?**

As you already know, your child is suffering from tuberculosis disease. As it was explained to you before, tuberculosis is a infectious disease and there is an RNTCP policy to give anti TB treatment in a directly observed treatment by the DOT Provider which may be of Government or Community trained DOT Provider to ensure regular treatment for better cure from the disease, Generally the treatment duration is around 6 to 8 months and if patient complete this treatment it is very likely that he/she will be cured

**What is the intervention in this study?**

As per this study, we will compare children whose treatment is administered and observed by family members with those who get their treatment from other people, to see if the progress and outcome are the same or different.

A family member will be selected as the DOT Provider to give medicine to child (0 to 14 years) which will last for 6-7 months. Family member will be given training at nearest health facility on how to give the drugs to the child, and about assessment of side effects during the treatment if any. Treatment card should be updated regularly by the selected family DOT Provider.

**What are the possible side effects of this treatment and study?**

These drugs have some possible side effects including nausea, vomiting, lack of appetite, jaundice, and ringing in the ears etc. However your doctor of concerned PHI, Health worker and trained community DOT provider of your area together with the whole team, will closely take care of your child during treatment help you to give advice to overcome these side effects.

There are not any foreseen adverse events anticipated by participation/non participation in the study. Clinical assessment & Treatment of child will be free-of-charge irrespective whether family member is DOT provide or not.

**How the government system will monitor my child therapy?**

Throughout treatment of your child there will be a team of Heath & RNTCP staff that will monitor your progress

Medical officer of PHI will regularly assess clinically to your child related to health and how treatment is progressing. This information will then be discussed and analyzed and presented to authorities in order to help the doctors find out that the better DOT strategy, and what are the things they can do to improve it. No one will have access to any information and name other than the PHI staff. All information given to authorities or medical professionals in India and elsewhere in the world will not contain names or other signs that can identify your child.

**What will happen to information collected in the study?**

Participation in the study and all information collected will remain confidential to the principle investigator and while disseminating the study findings to the external audience, only pooled/aggregate results will be shared.

**What will happen if we refuse to participate in the study?**

If you decide not to participate in this study, this will have no negative consequence on the way your child treated now. Your child will have the opportunity to receive the same TB treatment from either Government or Community DOT provider as the rest of the patients. Doctor of concerned PHI, Health worker and trained community DOT provider of your area together with the whole team, will closely take care of your child during entire treatment period.

**Where can I get more information?**

If you have any questions, please contact:

Dr. P. V. Dave, Adl Director, Public Health, Dept of Health & Family Welfare, Government of Gujarat. Phone No: 079-23253330 Fax No: 079-23253559 email ID: stogu@rntcp.org Mob no: 09376176580
