# Supplementary material for: Direct Observation of Treatment Provided by a Family Member as Compared to Non-Family Member among Children with New Tuberculosis: A Pragmatic, Non-Inferiority, Cluster-Randomized Trial in Gujarat, India
Source: PLoS One. 2016 Feb 5;11(2):e0148488. doi: 10.1371/journal.pone.0148488 (PMC4743945; doi:10.1371/journal.pone.0148488)
Supplement: S1 Protocol — (ZIP) [file pone.0148488.s004.zip › approved pediatric or protocol/01 Annexure 6 Assent form.doc]

|  | **Family DOT study**  **Assent form (applicable to children old enough to understand and be able to agree or not to participation)** |
| --- | --- |

**Project title: Prospective study on inclusion of Family member as a DOT provider for Child TB Patient in State of Gujarat**

| **Name of Principal investigator** | Dr Paresh Dave |
| --- | --- |
| **Address of principal investigator** | Department of Health & Family Welfare, Dr. Jivaraj Mehta Bhavan, Block-5, Government of Gujarat, Gandhinagar, Phone-079-23253330. |
| **Email address of principal investigator** | [stogu@rntcp.org](mailto:stogu@rntcp.org) |

We are doing a study to learn about who should be treatment provider for children suffering from tuberculosis.

If you agree to be in our study, we will keep you in the study and as per your choice any of adult family member can be your treatment provider. If it is objectionable to you than health worker from the government side will provide treatment to you.

You can ask questions about this study at any time. If you decide at any time not to finish, you can ask us to stop.

If you sign this paper, it means that you have read this and that you want to be in the study. If you don’t want to be in the study, don’t sign this paper. Being in the study is up to you, and no one will be upset if you don’t sign this paper or if you change your mind later.

Your signature: ____________________________________Date___________

Your printed name: _________________________________Date___________

Signature of person obtaining consent: _________________Date____________

Printed name of person obtaining consent: _______________Date___________
